# Prudent Use of Antibiotics in Dairy Cows: The Nordic Approach to Udder Health

**DOI:** 10.3389/fvets.2021.623998

**Published:** 2021-03-05

**Authors:** Päivi Rajala-Schultz, Ane Nødtvedt, Tariq Halasa, Karin Persson Waller

**Affiliations:** ^1^Department of Production Animal Medicine, Faculty of Veterinary Medicine, University of Helsinki, Helsinki, Finland; ^2^Department of Production Animal Clinical Sciences, Faculty of Veterinary Medicine, Norwegian University of Life Sciences, Ås, Norway; ^3^Section of Welfare and Disease Control, Faculty of Medical and Health Sciences, Institute of Veterinary and Animal Sciences, University of Copenhagen, Copenhagen, Denmark; ^4^Department of Animal Health and Antimicrobial Strategies, National Veterinary Institute (SVA), Uppsala, Sweden

**Keywords:** mastitis control, bovine, antibiotic use, therapy, bacteriologic diagnosis

## Abstract

Global concerns regarding bacterial antibiotic resistance demand prudent use of antibiotics in livestock production. Dairy production in the Nordic countries has a low consumption of antibiotics, while animal health, productivity and milk quality are at high levels. Here, we describe the basis of Nordic mastitis control and treatment strategies, as a model for production of high-quality milk with prudent use of antibiotics. We hope this will be beneficial for dairy producers and advisors in other countries and regions that consider limiting antibiotic use in cattle herds. In this perspectives paper we describe the dairy sector in the Nordic countries, and present regulatory aspects of antibiotic use, diagnostics and current guidelines for treatment of clinical mastitis as well as dry cow therapy. We also show summary statistics of udder health indicators in Denmark, Finland, Norway and Sweden, to illustrate the effects of the implemented udder health management practices.

## Introduction

Antibiotic resistance is a global concern because of its fast spread not only in human, but also in animal populations (1–3). A common feature for livestock production in the Nordic countries is the constant focus on prudent use of antibiotics. The overall consumption of antibiotics in animal populations in Denmark, Finland, Iceland, Norway and Sweden is the lowest among European countries, as measured by mg of active ingredients per kg of estimated biomass of animal populations (4). Reports on sales of veterinary antimicrobial agents in European countries are published regularly by the European Medicines Agency (EMA) and comparative sales figures can be found on these reports. Moreover, the tradition of prudent antibiotic use in production animals goes back several decades and has contributed to low levels of antibiotic resistance in bacteria isolated from this sector across the Nordic countries (5, 6). In general, antibiotic resistance is quite uncommon in dairy cattle in the region, compared to that in other species and regions.

Dairy production has long traditions in the region and stakeholders across the country borders collaborate actively on various issues. As an example, joint Nordic guidelines for treatment of mastitis in dairy cows were agreed upon in a consensus meeting initiated by the Nordic collaborative group of dairy processors and published in 2009 (7). Generally, initiation of antibiotic treatment for intramammary infections is expected to be based on microbiologic diagnosis and benzylpenicillin is the drug of choice in most cases. Prophylactic use of antibiotics is discouraged in all production animals in the region.

The aim of this paper is to describe the basis of Nordic mastitis control and treatment strategies, as a model for high-quality milk production with prudent use of antibiotics. The focus will be on data from Denmark, Finland, Norway and Sweden. We describe the dairy sector in the region, regulatory aspects of antibiotic use, diagnostics and current guidelines for treatment of clinical mastitis as well as for dry cow therapy. Further, we will present summary statistics regarding udder health indicators to demonstrate some outcomes of the existing control and treatment strategies.

## Structure of Nordic Dairy Production

Dairy farming in the Nordic countries is characterized by small, family-run farms. National dairy herd improvement organizations collect data on milk production, milk components and milk somatic cell counts (SCC) and provide support also on nutrition and farm economics. The dairy herd milk recording schemes have a high coverage, which ranges from approximately 80 to over 90% of the cows within each country. Additionally, information on veterinary-diagnosed and -treated diseases is recorded and collected from all farms to a centralized national database in each Nordic country. Data collection and recording is at present largely electronic. In the Nordic countries, producers do not have access to veterinary drugs without a prescription from a veterinarian and thus most treatments are initiated by veterinarians who are also expected to record them. While the disease recording is not 100% complete (8, 9), the system has quite high accuracy and is unique on a global scale. Additionally, a coding scheme for the veterinary diagnoses has been jointly agreed upon and as a result, health and production are well-monitored in the Nordic region and at the national levels. The national dairy organizations are mostly farmer-owned co-operatives, and this contributes to a good climate for collaboration where farmers, veterinarians, researchers and legislators strive together for good milk quality and animal health. There is high trust and confidence among the different stakeholders, who work closely together when developing new recommendations and regulations on issues such as antibiotic use. The goal is to ensure that: (1) the recommendations and regulations are evidence-based and (2) they have high level of acceptability and implementation.

As can be seen in Table 1, the average herd size varies across the region with Danish herds being the largest and Norwegian the smallest. However, herd sizes are increasing and number of herds decreasing every year in all four countries, in keeping with the global trend. Automatic milking systems (AMS) have become common in all the Nordic countries since the introduction in 1989, and 30% of all milk produced in the region is estimated to come from cows milked in AMS herds (10). Small tie-stall barns are still common, but their numbers are constantly decreasing and new facilities in all four countries are free-stall barns, often with AMS. The average milk yield per cow in the region is highest in Denmark and lowest in Norway. This is partially explained by genetics, because Danish Holstein is the dominating breed in Denmark, while the combined milk- and meat-producing Norwegian Red dominates its homeland. In addition to focusing on productivity, breeding programs in the Nordic countries have also long focused on health, including resistance to mastitis (11).

**Table 1:** Descriptive statistics of milk production of dairy cows in four Nordic countries.

	**Denmark^**a**^**	**Finland^**b**^**	**Norway^**c**^**	**Sweden^**d**^**
Dairy herds, number	2 715	5 783	7 831	3 253
Average herd size (milking cows)	213	48	28	94
Proportion (%) herds with automatic milking systems^a^	25	20	23	28
Average annual milk yield, kg ECM^e^/cow	11 037	10 534	8 602	10 417

a *Data obtained from January 2020 statistics (SEGES (an agricultural knowledge & innovation center), Denmark, www.seges.dk)*.

b *Number of herds and herd size in December 2019, yield data for herds in milk recording scheme during 2019*.

c *Number of herds and herd size 2019 (Norwegian Agriculture Agency; www.landbruksdirektoratet.no), yield data for herds in dairy herd recording system*.

d *Number of herds and herd size 2019 (National Board of Agriculture; www.jordbruksverket.se), yield data for herds in milk recording scheme during 2019*.

e *ECM = energy-corrected milk, accounts for the variability in milk fat and protein contents*.

## Regulations and Recommendations on Antibiotic Use

Legislation in the Nordic countries allows the purchase of antibiotics for use in animals only based on a prescription from a veterinarian. In addition, antibiotics should not be used for prophylactic purposes or as growth promoters. Administration of antibiotics may, however, be carried out by a farmer as instructed by a herd veterinarian, depending on the level of the advisory agreement between them. The sale of antibiotics is generally only through pharmacies or directly from veterinarians who are not allowed to make any profit from these sales. Veterinary use of critically important antibiotics is strongly discouraged across the region. Veterinary and farm records of antibiotic sales and usage can be randomly checked by the authorities for compliance. For example, Nordic countries do not allow routine prophylactic antibiotic use at dry-off and in some countries farmers can be fined, if found to be breaking the law. Similarly, veterinarians could lose their licenses, temporarily or permanently, if their antibiotic prescribing practices are repeatedly found in violation of the regulations, but this happens rarely.

Udder health experts in each country have developed guidelines on treatment of mastitis of dairy cows. These guidelines complement the legal regulations regarding dispensing of antibiotics and provide practical guidance regarding the use of antibiotics in mastitis therapy. They vary to some degree between the Nordic countries due to differences in legislation, availability of drugs, distribution of mastitis-causing pathogens and their susceptibility to antibiotics, national studies, tradition and policies.

## Diagnostics of Bacteriology and Antibiotic Resistance

Bacteriologic diagnosis is an important part of the Nordic guidelines for mastitis therapy (7). Knowledge regarding aetiologic agents, patterns of udder infections and antibiotic resistance in a herd is essential when choosing the best treatment and control measures. This approach is a part of the basic veterinary training in the Nordic countries and the same message of prudent antibiotic usage is also conveyed to dairy farmers. To obtain this information, milk samples should be taken before treatment decisions, and sent to a laboratory for microbiologic analysis. Culturing of milk samples at veterinary clinics using selective agar plates can also be of value to quickly confirm the drug of choice in acute cases of clinical mastitis. Bacteriologic follow-up after treatment might also be of interest in some cases.

A veterinarian or a farmer typically collects milk samples, which are transported to the laboratory via postal mail or in a milk truck of a dairy co-operative, as some dairy co-operatives have their own diagnostic mastitis laboratories. In acute clinical cases of mastitis, treatment is often given immediately and adjusted as needed when bacteriologic results become available. In most mastitis cases, whether clinical or subclinical, producers take milk samples for testing, partly hoping that use of antibiotics and consequently discarding of milk could be avoided based on the causal agent, or lack thereof. To date, bacteriologic culturing is the most common method in all countries except in Finland where most milk samples are analyzed using polymerase chain reaction (PCR) technology. Presence of beta-lactamase production in staphylococci is routinely investigated. Evaluation of resistance against other antibiotics or among other bacteria can be performed if needed.

Good knowledge on and monitoring national trends in occurrence of mastitis-causing pathogens and their antibiotic resistance is important when forming recommendations for treatment and control of mastitis. At present, no common Nordic scheme exists for such monitoring, but several studies on clinical or subclinical mastitis have been performed at national levels (12–19). Compiled annual laboratory data are also available (20, 21). Overall, the most common micro-organisms found in association with bovine mastitis in the Nordic countries are staphylococci and streptococci. Streptococci are mostly sensitive to penicillin, as are the majority of the staphylococci although this varies somewhat between staphylococcal species and countries. Contrary to many other countries (22, 23), Gram-negative bacteria, such as *Escherichia coli*, are of lesser importance.

## Treatment Guidelines

According to the Nordic guidelines for mastitis therapy, decisions on antibiotic treatment should be based on evaluation of prognosis and bacteriologic diagnosis (7). Moreover, the use of antibiotics during lactation should primarily be considered for cases of acute clinical mastitis. Antibiotic treatment of subclinical mastitis should mainly be done at dry-off.

### Clinical Mastitis

In clinical mastitis, benzylpenicillin is the drug of choice, unless the causative pathogen is a Gram-negative bacterium or known to be resistant to penicillin. In such cases, mostly supportive therapy is recommended. According to the guidelines, treatment length when using benzylpenicillin varies between 3 and 5 days depending on the pathogen, e.g., for S*taphylococcus aureus* and *Streptococcus uberis* IMI a 5-d treatment is recommended, but for non-aureus staphylococcal (NAS) IMI a shorter therapy is often considered adequate. Recommendations on the route of administration, however, are not given at the Nordic level. Various combinations of local and/or systemic treatments are applied in each country. In all cases, supportive measures (e.g., sick pen, optimal cow comfort) and supportive therapy (e.g., NSAID, fluid therapy) as well as biosecurity actions (e.g., segregation) should always be considered. In a survey, two-thirds of Swedish veterinarians reported using NSAIDs always or almost always when treating clinical mastitis (24).

### Dry Cow Therapy

As mentioned above, the Nordic guidelines for mastitis therapy mention the possibility to treat subclinical mastitis with antibiotics at dry-off, but they provide no further details or advice on the practice. The use of dry cow therapy (DCT) has been an important part of most mastitis control programs, but its implementation differs among regions of the world. In many countries, blanket DCT, i.e., treating all quarters of all cows with antibiotics at dry-off, regardless of their infection status, has long been recommended and used (25–28). Currently, however, due to the growing concerns about antibiotic resistance, selective DCT is being studied and considered worldwide in herds with low levels of contagious mastitis problems (29–32). In the Nordic countries, the recommendation has always been to implement selective DCT, i.e., to treat only infected cows (33–36). Selection of cows that are candidates for DCT is mostly done based on SCC data from 1 to 3 milk recordings before drying-off, data from the AMS system, mastitis history and possibly California Mastitis Test (CMT) scores. In all Nordic countries, having a bacteriologic diagnosis is encouraged before antibiotic therapy at dry-off is initiated, or at least knowing the pathogen profile and susceptibility of mastitis-causing pathogens in the herd. For example, if a causal agent of a subclinical IMI was detected earlier during the lactation, but treatment was postponed until dry-off, a new sampling of the cow might not be performed prior to DCT. The focus is to mainly treat penicillin-susceptible intramammary infections using long-acting benzylpenicillin. Penicillin-resistant NAS infections may be treated with cloxacillin-containing products and with older, chronically infected cows, culling is often recommended in the short- to medium-term.

## Trends in Udder Health Indicators

**Figure 1:**
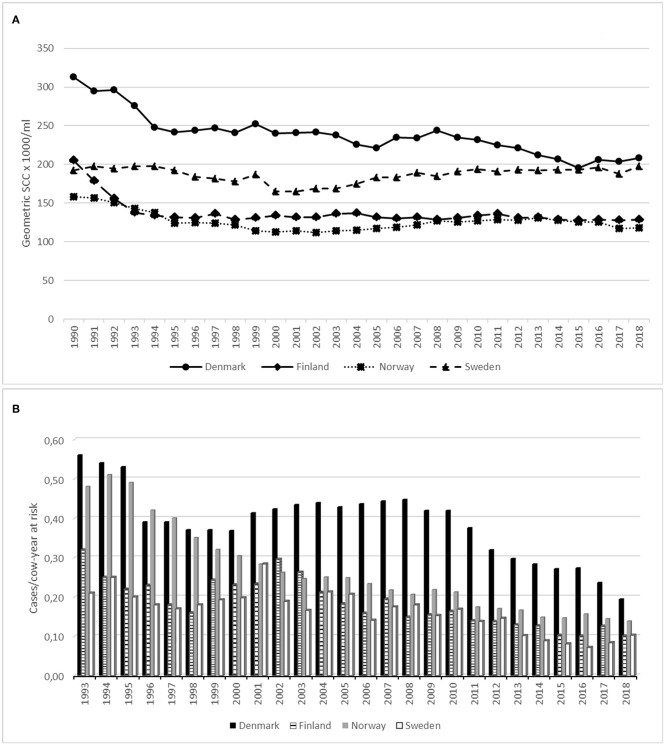
Geometric mean of bulk tank milk somatic cell count (SCC) (cells × 1,000/mL) from 1990 to 2018 **(A)** and incidence rate of clinical mastitis* (cases/cow year at risk) from 1993 to 2018 **(B)** in four Nordic countries. *All treatments are administered or initiated by a veterinarian.

Data on udder health indicators compiled by the collaborative group of Nordic dairy processors are presented in Figure 1 (modified from (37)). The figures show a decreasing trend both in the incidence of clinical mastitis and in SCC during the period from around early 1990-ies until today. Geometric means of bulk tank milk SCC are currently approximately 200,000 cells/ml in Denmark and Sweden and around 125,000 cells/mL in Finland and Norway. The incidence rates for treatment of clinical mastitis in 2018 were 0.19 and 0.10 per cow year in Denmark and Sweden, respectively. The trend is toward fewer treatments of clinical mastitis across the region during this time-period. As an example, the treatment frequency for clinical mastitis in Norway decreased by 4.2% from 2017 to 2018, while the reduction from 1994 to 2018 was 73% (20) (Figure 1). In other European countries with major dairy production, such as The Netherlands, bulk tank SCC is relatively similar to that in the Nordic countries; a mean value of 171,000 cells/mL. However, incidence of clinical mastitis (28.6 cases per 100 cows-year) and frequency of antibiotic treatments were higher than those in the Nordic countries (38).

Overall, the use of antibiotic DCT is low in the Nordic countries, with an estimated one-third or less of cows receiving such treatment at the end of lactation. According to a recent survey of DCT routines, 78% of Finnish producers report using selective DCT and 9% no DCT at all. The remaining proportion of herds reported treating all cows at dry-off and these herds were typically larger and more frequently had an automatic milking system compared to the other groups. In the majority (71%) of the selective DCT herds, less than one-fourth of the cows receive DCT at dry-off (39). In a similar study in Sweden, 96% of the farmers said they use selective DCT and most of those treat 25% or less of the cows (Persson Waller et al., unpublished). Information from Norway indicates that the treatment frequency for DCT there is even lower (O. Østerås, personal communication).

## Discussion

In a time when antibiotic resistance is recognized as a threat to animal and human health (3), food animal producers and veterinarians must continue to strive for prudent antibiotic use and sustainable production. This is an obvious One Health challenge and all stakeholders, industry in the front row, must actively participate. If consumers do not find animal-derived food sustainably and ethically produced, demand and markets for these products will likely shrink. In fact, market demands and consumer concerns e.g., on animal welfare and antibiotic resistance can be driving forces for changes in routines and procedures used in animal production (40, 41).

In the Nordic region, strong trust among farmers, consumers, educators, researchers and governmental agencies has enabled introduction and implementation of both strict legislation and recommendations on antibiotic use in animal production. This approach is widely embraced in the society and the recommendations rely on the willingness of all stakeholders to cooperate. This, as well as industry initiatives, have resulted in a marked decrease in antibiotic use across the Nordic countries during the past decades. Simultaneously, milk SCC levels and occurrence of clinical mastitis have decreased in most countries or remained stable. Production and health parameters have been recorded at cow level in the Nordic countries for decades, mainly for breeding reasons, but also to monitor health. These data have played a central role in establishing control and/or eradication programs for different diseases in the region, including mastitis. High quality milk production is also a result of this tradition.

It should be noted that dairy herd sizes in the Nordic countries are small when compared to other regions with major milk production. This has likely contributed to the current situation with low antibiotic usage and good animal health. It is also important to note that while veterinarians and producers in many other countries and regions are now adjusting to a new situation where antibiotic usage is becoming more regulated, the Nordic counterparts have always lived in that situation and consider it the norm. However, herd sizes are currently increasing everywhere, also in the Nordic countries. Optimal management and continued monitoring of animal health, milk quality and antibiotic consumption will be key elements in maintaining our favorable situation even as herds grow larger. Up-to-date knowledge of causal agents of intramammary infections, especially in free-stall and AMS farms will be crucially important in larger herds. This will assist in preventing and rapidly controlling potential spread of contagious pathogens, such as *Streptococcus agalactiae*. This pathogen had been eradicated decades ago from all Nordic countries, but it has recently re-emerged in some Nordic dairy herds, and interestingly, it is displaying also a potential feco-oral transmission routes (42).

We hope that the Nordic approach to dairy production might serve as an inspiration so that bacteriologic diagnosis of mastitic milk samples before initiation of antibiotic treatments, use of narrow-spectrum antibiotics and selective DCT can become the norm also in other regions. The Nordic experience provides evidence that prudent use of antibiotics is possible, without sacrificing animal health or milk quality. Continuous education of veterinarians and producers is pivotal to maintain the favorable situation in the region. In addition to veterinarians, farmers and legislators, it is also important that the pharmaceutical industry understands this strategy and ensures availability of suitable, narrow-spectrum antibiotics.

In conclusion, the Nordic experience shows that it is possible to maintain low incidence of clinical mastitis and acceptable SCC levels with prudent use of antibiotics and selective DCT based on bacteriologic diagnosis of intramammary infections in a region with high milk production.

## Data Availability Statement

The raw data supporting the conclusions of this article will be made available by the authors, without undue reservation.

## Author Contributions

All authors have equally contributed to the planning, writing and editing of the manuscript.

## Conflict of Interest

The authors declare that the research was conducted in the absence of any commercial or financial relationships that could be construed as a potential conflict of interest.

## References

[1] Birkegard AC Halasa T Toft N Folkesson A Graesboll K. Send more data: a systematic review of mathematical models of antimicrobial resistance. Antimicrob Resist In. (2018) 7:117. 10.1186/s13756-018-0406-1 PMC6162961 30288257

[2] OIE. The OIE Strategy on Antimicrobial Resistance and the Prudent Use of Antimicrobials. (2016). Available online at: https://www.oie.int/fileadmin/Home/eng/Media_Center/docs/pdf/PortailAMR/EN_OIE-AMRstrategy.pdf (accessed January 7, 2021). 10.20506/bull.2016.3.2557

[3] WHO. No Time to Wait: Securing the Future From Drug-Resistant Infections. (2019). Available online at: https://www.who.int/antimicrobial-resistance/interagency-coordination-group/IACG_final_report_EN.pdf?ua=1/ (accessed January 7, 2021).

[4] EMA E. Sales of Veterinary Antimicrobial Agents in 31 European Countries in 2017. Trends from 2010 to 2017 2019. Contract No.: EMA/294674/2019.

[5] EFSA ECDC. The European Union summary report on antimicrobial resistance in zoonotic and indicator bacteria from humans, animals and food in 2015. EFSA J. (2017) 15:4694. 10.2903/j.efsa.2017.4694 PMC7009883 32625402

[6] EFSA ECDC. The European Union Summary Report on Antimicrobial Resistance in zoonotic and indicator bacteria from humans, animals and food in 2017/2018. Efsa J. (2020) 18:6007. 10.2903/j.efsa.2020.6007 PMC7448042 32874244

[7] Anon. Nordic guidelines for mastitis therapy Lund, Sweden. (2009). Available online at: https://old.sva.se/globalassets/redesign2011/pdf/antibiotika/antibiotikaresistens/nordic-guidelines-for-mastitis-therapy.pdf (accessed January 7, 2021).

[8] Wolff C Espetvedt M Lind AK Rintakoski S Egenvall A Lindberg A. Completeness of the disease recording systems for dairy cows in Denmark, Finland, Norway and Sweden with special reference to clinical mastitis. Bmc Vet Res. (2012) 8:131. 10.1186/1746-6148-8-131 22866606 PMC3489834

[9] Espetvedt MN Rintakoski S Wolff C Lind AK Lindberg A Virtala AM. Nordic veterinarians' threshold for medical treatment of dairy cows, influence on disease recording and medicine use: mild clinical mastitis as an example. Prev Vet Med. (2013) 112:76–89. 10.1016/j.prevetmed.2013.07.004 23948145

[10] Sigurdsson S Hettasch T Gretarsson S Kromann H Manninen E Nyman K. Development of AMS in the Nordic countries between 1998 and 2018. IDF Mastitis Conference; May 14-16, 2019. Copenhagen, Denmark (2019).

[11] Heringstad B Klemetsdal G Ruane J. Selection for mastitis resistance in dairy cattle: a review with focus on the situation in the Nordic countries. Livest Prod Sci. (2000) 64:95–106. 10.1016/S0301-6226(99)00128-1

[12] Myllys V Asplund K Brofeldt E Hirvela-Koski V Honkanen-Buzalski T Junttila J. Bovine mastitis in Finland in 1988 and 1995 - changes in prevalence and antimicrobial resistence. Acta Vet Scand. (1998) 39:119–26. 10.1186/BF03547813 9592952 PMC8050697

[13] Pitkala A Haveri M Pyorala S Myllys V Honkanen-Buzalski T. Bovine mastitis in Finland 2001 - prevalence, distribution of bacteria, and antimicrobial resistance. J Dairy Sci. (2004) 87:2433–41. 10.3168/jds.S0022-0302(04)73366-4 15328265

[14] Bengtsson B Unnerstad HE Ekman T Artursson K Nilsson-Ost M Waller KP. Antimicrobial susceptibility of udder pathogens from cases of acute clinical mastitis in dairy cows. Vet Microbiol. (2009) 136:142–9. 10.1016/j.vetmic.2008.10.024 19058930

[15] Ericsson Unnerstad H Lindberg A Persson Waller K Ekman T Artursson K Nilsson-Ost M. Microbial aetiology of acute clinical mastitis and agent-specific risk factors. Vet Microbiol. (2009) 137:90–7. 10.1016/j.vetmic.2008.12.005 19155148

[16] Persson Y Nyman AK Gronlund-Andersson U. Etiology and antimicrobial susceptibility of udder pathogens from cases of subclinical mastitis in dairy cows in Sweden. Acta Vet Scand. (2011) 53:36. 10.1186/1751-0147-53-36 21649936 PMC3118135

[17] Vakkamaki J Taponen S Heikkila AM Pyorala S. Bacteriological etiology and treatment of mastitis in Finnish dairy herds. Acta Vet Scand. (2017) 59:33. 10.1186/s13028-017-0301-4 28545485 PMC5445452

[18] Chehabi CN Nonnemann B Astrup LB Farre M Pedersen K. *In vitro* antimicrobial resistance of causative agents to clinical mastitis in Danish dairy cows. Foodborne Pathog Dis. (2019) 16:562–72. 10.1089/fpd.2018.2560 31059284

[19] Ericsson Unnerstad H Waldner J Persson Waller K. Bacterial Findings at Clinical Mastitis in Swedish Dairy Cows. IDF mastitis conference May 14-16, 2019. Copenhagen, Denmark (2019). p. 18.

[20] Osterås O. Helsekortordningen, storfe 2019 – Statistikksamling. Ås (2020). [Health recordings in dairy cattle, 2019] Available online at: https://medlem.tine.no/fagprat/husdyrkontrollen/_attachment/499149?_ts=170afd0cd3d (accessed January 7, 2021).

[21] Anon. Redogörelse för husdjursorganisationernas Djurhälsovård 2018/2019 [Livestock organizations' Animal healthcare report 2018/2019]. (2020). Available online at: https://www.vxa.se/globalassets/dokument/statistik/redogorelse-for-husdjursorganisationernas-djurhalsovard-2018-2019.pdf (accessed January 7, 2021).

[22] Bradley AJ Leach KA Breen JE Green LE Green MJ. Survey of the incidence and aetiology of mastitis on dairy farms in England and Wales. Vet Rec. (2007) 160:253–8. 10.1136/vr.160.8.253 17322356

[23] Oliveira L Hulland C Ruegg PL. Characterization of clinical mastitis occurring in cows on 50 large dairy herds in Wisconsin. J Dairy Sci. (2013) 96:7538–49. 10.3168/jds.2012-6078 24119795

[24] Persson Waller K Hardemark V Nyman AK Duse A. Veterinary treatment strategies for clinical mastitis in dairy cows in Sweden. Vet Rec. (2016) 178:240. 10.1136/vr.103506 26864025

[25] Neave FK Dodd FH Kingwill RG Westgarth DR. Control of mastitis in the dairy herd by hygiene and management. J Dairy Sci. (1969) 52:696–707. 10.3168/jds.S0022-0302(69)86632-4 5391057

[26] Bertulat S Fischer-Tenhagen C Heuwieser W. A survey of drying-off practices on commercial dairy farms in northern Germany and a comparison to science-based recommendations. Vet Rec Open. (2015) 2:e000068. 10.1136/vetreco-2014-000068 26392891 PMC4567148

[27] USDA 2016. Dairy 2014, Dairy Cattle Management Practices in the United States. Fort Collins, CO: USDA–APHIS–VS–CEAH–NAHMS. Fort Collins, CO (2014).

[28] Fujiwara M Haskell MJ Macrae AI Rutherford KMD. Survey of dry cow management on UK commercial dairy farms. Vet Rec. (2018) 183:1–8. 10.1136/vr.104755 29907660

[29] Cameron M McKenna SL MacDonald KA Dohoo IR Roy JP Keefe GP. Evaluation of selective dry cow treatment following on-farm culture: risk of postcalving intramammary infection and clinical mastitis in the subsequent lactation. J Dairy Sci. (2014) 97:270–84. 10.3168/jds.2013-7060 24183691

[30] Scherpenzeel CGM Tijs SHW den Uijl IEM Santman-Berends I Velthuis AGJ Lam T. Farmers' attitude toward the introduction of selective dry cow therapy. J Dairy Sci. (2016) 99:8259–66. 10.3168/jds.2016-11349 27448856

[31] Vasquez AK Nydam DV Foditsch C Wieland M Lynch R Eicker S. Use of a culture-independent on-farm algorithm to guide the use of selective dry-cow antibiotic therapy. J Dairy Sci. (2018) 101:5345–61. 10.3168/jds.2017-13807 29605332

[32] Rowe SM Godden SM Nydam DV Gorden PJ Lago A Vasquez AK. Randomized controlled trial investigating the effect of 2 selective dry -cow therapy protocols on udder health and performance in the subsequent lactation. J Dairy Sci. (2020) 103:6493–503. 10.3168/jds.2019-17961 32331877

[33] Bratlie O. Dry cow therapy. Vet Rec. (1973) 93:430–1. 10.1136/vr.93.15.430 4797888

[34] Østerås O Sandvik L. Effects of selective dry-cow therapy on culling rate, clinical mastitis, milk yield and cow somatic sell count. A randomized clinical field study in cows. J Vet Med. (1996) 43:555–75. 10.1111/j.1439-0450.1996.tb00353.x 8976620

[35] Ekman T Østerås O. Mastitis control and dry cow therapy in the Nordic countries. 42nd Annual Meeting of National Mastitis Council. Fort Worth, Texas: National Mastitis Council (2003) p. 18–30.

[36] Rajala-Schultz P Persson Waller K Halasa T Nodtvedt A. Selective approach to dry cow therapy. Vet Rec. (2019) 184:29–30. 10.1136/vr.k5405 30606859

[37] Østerås O Landin H Kulkas L Farre M Reiersen J. The Udder Health in the Nordic Countries. IDF Mastitis Conference. Copenhagen, Denmark: IDF (2019). p. 52–6.

[38] Santman-Berends I Swinkels JM Lam T Keurentjes J van Schaik G. Evaluation of udder health parameters and risk factors for clinical mastitis in Dutch dairy herds in the context of a restricted antimicrobial usage policy. J Dairy Sci. (2016) 99:2930–9. 10.3168/jds.2015-10398 26874413

[39] Vilar MJ Hovinen M Simojoki H Rajala-Schultz PJ. Short communication: drying-off practices and use of dry cow therapy in Finnish dairy herds. J Dairy Sci. (2018) 101:7487–93. 10.3168/jds.2018-14742 29753489

[40] Boogaard BK Oosting SJ Bock BB Wiskerke JS. The sociocultural sustainability of livestock farming: an inquiry into social perceptions of dairy farming. Animal. (2011) 5:1458–66. 10.1017/S1751731111000371 22440292

[41] Loria K. McDonald's Announces Plan to Reduce Antibiotic Use in Beef. Consumer Report. (2018). Available online at: https://www.consumerreports.org/food-safety/mcdonald-s-announces-plan-to-reduce-antibiotics-in-beef/ (accessed January 7, 2021).

[42] Jorgensen HJ Nordstoga AB Sviland S Zadoks RN Solverod L Kvitle B. Streptococcus agalactiae in the environment of bovine dairy herds - rewriting the textbooks? Vet Microbiol. (2016) 184:64–72. 10.1016/j.vetmic.2015.12.014 26854346

